# Live, attenuated rubella vectors expressing HIV and SIV vaccine antigens

**DOI:** 10.1186/1742-4690-9-S2-P1

**Published:** 2012-09-13

**Authors:** I Berkower, Y Ni, K Virnik

**Affiliations:** 1 Center for Biologics, U.S. Food and Drug Administration, Bethesda, MD, USA

## Background

Despite progress in identifying targets of neutralizing antibodies and T cell immunity, many HIV antigens have been weak immunogens. We have developed rubella as a live viral vector to enhance immunogenicity by presenting HIV and SIV antigens in the context of an acute infection. These vectors are based on the rubella vaccine strain RA27/3: safety and immunogenicity have been established in millions of children around the world. If the vector loses its insert, it would revert to the vaccine strain. It is immunogenic: one dose protects for life (against rubella). It elicits mucosal and systemic immunity. Rubella readily infects rhesus macaques, and these animals can test vector growth, immunogenicity and protection against SIV or SHIV challenge.

## Methods

Starting from the live, attenuated vaccine strain, we have identified two insertional sites, where foreign genes can be expressed without compromising rubella protein expression or titer.

## Results

Rubella can accommodate foreign inserts at either of two sites. Inserts at the nonstructural site were expressed as a fusion protein with rubella nonstructural protein p150. At the structural site, inserts were expressed with the structural polyprotein, processed to free protein, and incorporated into virions. Foreign antigens, including GFP, SIV Gag, and HIV MPER, were stably expressed for >10 passages. Six recombinant rubella vectors were selected for testing in vivo. Two of these grew well, infecting 3 out of 3 macaques while expressing HIV or SIV antigens.

## Conclusion

Live, attenuated rubella vectors provide a good vaccine platform for evaluating SIV and HIV antigens in vivo and comparing prime/boost strategies for immunogenicity and protection. The results may also apply to man, since the same vectors grow well in human cells and could be substituted for current rubella vaccines, or they could be given prior to the age of natural rubella infection (9 years).

